# A Comprehensive 16-Year Analysis of National Center for Health Statistics Data on the Top Three Causes of Death Before Age 75 by Sex, Race, and Hispanic Origin

**DOI:** 10.7759/cureus.49340

**Published:** 2023-11-24

**Authors:** Okelue E Okobi, Patra C Ezeamii, Victor C Ezeamii, Oluwatosin B Iyun, Tricia O Okoye, Elochukwu U Nwachukwu, Prosper I Oghenebrume

**Affiliations:** 1 Family Medicine, Larkin Community Hospital Palm Springs Campus, Miami, USA; 2 Family Medicine, Medficient Health Systems, Laurel, USA; 3 Family Medicine, Lakeside Medical Center, Belle Glade, USA; 4 Epidemiology and Public Health, Jiann-Ping Hsu College of Public Health, Georgia Southern University, Statesboro, USA; 5 Epidemiology and Public Health, School of Public Health and Family Medicine, University of Cape Town, Cape Town, ZAF; 6 Family Medicine, Ambrose Alli University, Ekpoma, NGA; 7 Family Medicine, University of Uyo Teaching Hospital, Uyo, NGA; 8 General Practice, Dnipropetrovsk Medical Academy, Dnipropetrovsk, UKR

**Keywords:** leading cause of death, years of potential life lost, sociodemographic variables, trend based analysis, national center for health statistics

## Abstract

Objective: This study aimed to conduct a comprehensive 16-year analysis of years of potential life lost (YPLL) due to leading causes of death in the United States, focusing on disparities by sex, race/ethnicity, and specific causes of death using the National Center for Health Statistics (NCHS) data.

Methods: Data from the NCHS spanning 2000-2016 were included. Age-adjusted YPLL rates per 100,000 population were analyzed, stratified by sex, race/ethnicity, and leading causes of death, including malignant neoplasms, heart disease, and cerebrovascular diseases.

Results: Over 16 years, the total YPLL rate was 7,036.2 per 100,000 population. Males had a higher YPLL rate (8,852.5 per 100,000) than females (5,259.9 per 100,000). Among racial/ethnic groups, Black/African Americans had the highest YPLL rate (10,896.8 per 100,000), followed by American Indian/Alaska Natives (7,310.0 per 100,000), Hispanics/Latinos (5,256.8 per 100,000), and Asians/Pacific Islanders (3,279.7 per 100,000). Leading causes included malignant neoplasms (1,451.6 per 100,000), heart diseases (1,055.4 per 100,000), and cerebrovascular diseases (182.3 per 100,000).

Conclusion: This analysis spanning 16 years highlights notable disparities in YPLL rates among different demographic groups. These differences are evident in the YPLL rates for males, which are higher than those for females. The YPLL rate is most pronounced among Black/African Americans, followed by American Indian/Alaska Natives, Hispanics/Latinos, and Asians/Pacific Islanders. The primary contributors to YPLL are malignant neoplasms, heart diseases, and cerebrovascular diseases. These findings emphasize the importance of addressing these disparities to enhance public health outcomes and mitigate the premature loss of life. Despite progress, disparities persist, highlighting the need for targeted interventions and further research.

## Introduction

Premature mortality, defined as death occurring before an individual's expected lifespan, remains a critical public health concern worldwide. It not only represents a profound loss of human potential but also poses substantial economic and societal burdens. One established measure for quantifying the impact of premature mortality is years of potential life lost (YPLL), which calculates the years that individuals could have lived if they had survived to their expected age of death. This measure provides valuable insights into the burden of various health conditions and helps prioritize public health interventions [1-3].

YPLL is a metric that estimates the years of life that individuals could have lived if they had not experienced premature death. By focusing on the potential years of life lost, YPLL offers a unique perspective on the burden of mortality, distinct from traditional measures that solely consider death counts or age-specific mortality rates. This metric enables public health professionals, policymakers, and researchers to better understand the societal impact of various causes of death and to prioritize interventions to reduce premature mortality effectively [3-6].

Heart disease, cerebrovascular diseases, and malignant neoplasms collectively account for a substantial portion of premature mortality in the United States. These diseases are not only major public health concerns but also represent conditions where preventive measures and early interventions can have a substantial impact. Understanding the epidemiological patterns of YPLL associated with these causes of death is crucial for targeted public health efforts [7-8].

This study focuses on the analysis of YPLL before the age of 75 (from birth to 75 years of life), categorized by sex, race, Hispanic origin, and the top three selected causes of death: heart disease, cerebrovascular diseases, and malignant neoplasms. The dataset spans a 16-year period, from 2000 to 2016, and leverages data from the National Center for Health Statistics (NCHS) data [9]. Analyzing YPLL due to specific causes of death is crucial for several reasons. First, it provides a quantitative measure of the impact of premature mortality on a population, offering insights into the overall health and well-being of communities. By identifying specific causes of death contributing to YPLL, public health officials can prioritize interventions and allocate resources more effectively. This study aims to add to the growing body of knowledge that analyzes the specific causes of premature mortality and their associated YPLL. By doing this, it hopes to improve some parts of our knowledge about the avoidable causes of early deaths, which will help public health plans and programs work to lower YPLL and make the health of the whole population better.

## Materials and methods

Study design

This comprehensive analysis aims to estimate YPLL due to leading causes of death over a 16-year period using data from the NCHS. The study employs a retrospective observational design to assess mortality data and calculate YPLL for various causes of death.

Data source and study population

The primary data source for this study is the National Vital Statistics System (NVSS) maintained by the NCHS. This database contains detailed information on deaths occurring in the United States, including cause of death, age at death, sex, and year of death. The study covers a 16-year period, from 2000 to 2016, to provide a comprehensive assessment of YPLL trends. The study includes all individuals whose deaths were recorded in the NVSS during the specified 16-year period. There are no age or sex restrictions, as the analysis aims to capture YPLL for leading causes of death across the entire population.

Outcome measures and variables

The primary outcome measure in this analysis is YPLL, a widely accepted indicator of premature mortality. YPLL is calculated by subtracting the age at death from a predefined standard age, typically 75 years, for each individual death. The total YPLL for a specific cause of death is obtained by summing the YPLL values for all individuals who died from that cause.

The key variables for this analysis include** **cause of death: data on the primary cause of death is categorized according to the International Classification of Diseases (ICD) codes, allowing for classification into specific causes, such as cardiovascular diseases, cancer, respiratory diseases, and others. The following ICD were used (all causes (ICD-10 code: A00-Y89), diseases of heart (ICD-10 code: I00-I09, I11, I13, I20-I51), ischemic heart disease (ICD-10 code: I20-I25), cerebrovascular diseases (ICD-10 code: I60-I69), malignant neoplasms (ICD-10 code: C00-C97), trachea, bronchus, and lung (ICD-10 code: C33-C34), colorectal (ICD-10 code: C18-C21), breast (ICD-10 code: C50), chronic lower respiratory diseases (ICD-10 code: J40-J47), influenza and pneumonia (ICD-10 code: J09-J18), chronic liver disease and cirrhosis (ICD-10 code: K70, K73-K74), diabetes mellitus (ICD-10 code: E10-E14), Alzheimer's disease (ICD-10 code: G30), human immunodeficiency virus (HIV) disease (ICD-10 code: B20-B24), unintentional injuries (ICD-10 code: V01-X59, Y85-Y86), motor vehicle-related injuries (ICD-10 code: V02-V04, V09.0, V09.2, V12-V14, V19.0-V19.2, V19.4-V19.6, V20-V79, V80.3-V80.5, V81.0-V81.1, V82.0-V82.1, V83-V86, V87, V89.0, V89.2), drug overdose (ICD-10 code: X40-X44, X60-X64, X85, Y10-Y14), nephritis, nephrotic syndrome, and nephrosis (ICD-10 code: N00-N07, N17-N19, N25-N27), suicide (ICD-10 code: X60-X84, Y87.0), homicide (ICD-10 code: X85-Y09). Sex: sex is recorded to assess potential variations in YPLL between males and females; racial pattern and year of death: the study covers a 16-year period, and the year of death is used to stratify the data for temporal analysis based on race and Hispanic origin.

Data analysis

Descriptive statistics were used to characterize the dataset, while temporal trends, stratified analyses, and inferential statistics were employed to extract meaningful insights. The study assessed how YPLL evolved over the 16-year period. The dataset was organized by year, and yearly YPLL totals were calculated for each cause of death, allowing for the identification of trends in premature mortality. Time series plots and line charts were employed to graphically represent these trends, aiding in the visualization of changes in premature mortality over time. Sex and race-based stratified analyses were also performed to assess potential sex disparities in YPLL patterns.

Ethical considerations

This study involves the analysis of de-identified mortality data, ensuring the privacy and confidentiality of individuals. Ethical approval is not required as the data is publicly accessible and does not involve human subjects.

## Results

The extensive 16-year analysis of NCHS data offers valuable insights into YPLL attributed to the leading causes of death in the United States. This comprehensive analysis covers a broad spectrum of causes, providing a thorough understanding of premature mortality trends. The subsequent section presents an in-depth overview of the significant findings from this analysis, as summarized in Table 1 below.

**Table 1:** Age-adjusted rate per 100,000 population under age 75 by sex, race, and Hispanic origin

Age-adjusted rate per 100,000 population under age 75 for sex, race, Hispanic origin		2000	2001	2002	2003	2004	2005	2006	2007	2008	2009	2010	2011	2012	2013	2014	2015	2016
	Total	7,578.1	7,531.9	7,521.7	7,473.1	7,280.8	7,315.7	7,228.7	7,087.0	6,957.7	6,833.1	6,642.9	6,635.2	6,588.0	6,593.1	6,622.1	6,757.7	6,968.6
Sex	Male	9,572.2	9,507.4	9,498.1	9,435.6	9,169.7	9,244.2	9,130.1	8,945.0	8,764.7	8,560.7	8,329.5	8,304.1	8,249.1	8,249.5	8,276.4	8,474.7	8,780.8
	Female	5,644.6	5,610.4	5,596.7	5,559.5	5,436.4	5,429.0	5,365.9	5,267.2	5,188.6	5,143.7	4,994.0	5,000.3	4,959.6	4,967.9	4,999.8	5,070.7	5,184.1
Race	White	6,949.5	6,950.4	6,962.7	6,931.1	6,775.0	6,823.2	6,763.4	6,664.3	6,590.9	6,486.5	6,342.8	6,365.7	6,321.6	6,338.2	6,390.1	6,514.8	6,715.1
	Black or African-American	12,897.1	12,569.6	12,463.1	12,311.4	11,872.4	11,788.4	11,515.2	11,098.2	10,611.2	10,319.6	9,832.5	9,659.5	9,555.7	9,528.5	9,490.6	9,702.3	10,030.4
	American Indian or Alaska Native	7,758.2	7,946.8	7,869.6	7,978.4	7,690.8	7,705.7	7,449.2	7,233.7	7,121.7	7,000.8	6,771.3	6,712.5	6,842.6	6,698.8	6,954.0	7,176.2	7,360.2
	Asian or Pacific Islander	3,811.1	3,726.5	3,598.0	3,564.5	3,371.7	3,433.5	3,361.3	3,262.2	3,154.7	3,114.2	3,061.2	3,014.4	3,049.4	3,050.9	2,954.4	3,049.7	3,176.8
	Hispanic or Latino	6,037.6	5,946.8	5,875.7	5,868.6	5,616.6	5,701.1	5,556.2	5,377.7	5,153.6	5,055.4	4,795.1	4,681.7	4,678.4	4,668.1	4,676.8	4,750.4	4,926.0

Based on sex

The study reveals significant variations in YPLL between males and females for various causes of death. Throughout the 16-year study period, the total YPLL rate was 7,036.2 per 100,000 population. When we break it down by sex, distinct patterns emerge. Males consistently exhibited a higher YPLL rate, with a male-specific YPLL rate of 8,852.5 per 100,000 population. In contrast, females reported a lower YPLL rate, at 5,259.9 per 100,000 population. Among males, the YPLL rate displayed fluctuations over the study period. It began at 9,572.2 per 100,000 population in 2000, gradually decreasing to 8,780.8 per 100,000 population in 2016 (with the lowest rate reported at 8,249.1 per 100,000 population in 2012). This reduction signifies a noteworthy improvement in premature mortality among males over the 16-year span.

**Figure 1:**
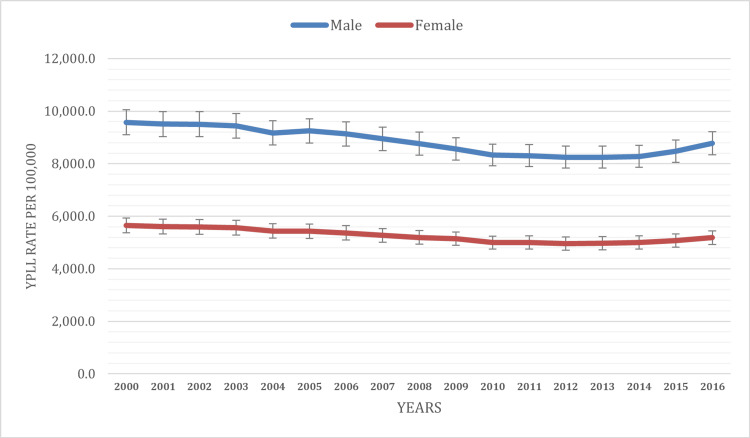
YPLL age-adjusted rate based on sex YPLL: Years of potential life lost

For females, the temporal trend in YPLL followed a similar pattern of decline, albeit with some variations. In 2000, the YPLL rate for females was 5,644.6 per 100,000 population, steadily decreasing over the years to reach 5,184.1 per 100,000 population in 2016 (with the lowest rate reported at 4,959.6 per 100,000 population in 2012) (Figure 1). This indicates a substantial enhancement in premature mortality rates among females during the study period. These disparities reflect differences in health behaviors, access to healthcare, and the prevalence of specific diseases between males and females.

Based on race

When we analyze the data by race, it becomes evident that the Black or African American population reported the highest YPLL rate during the study period, standing at 10,896.8 per 100,000 population. This was followed by the American Indian or Alaska Native population with a YPLL rate of 7,310.0 per 100,000 population, the Hispanic or Latino population with a rate of 5,256.8 per 100,000 population, and the Asian or Pacific Islander population with a rate of 3,279.7 per 100,000 population.

The YPLL rate for the White population exhibited a slight decline during the study period. Starting at 6,949.5 per 100,000 population in 2000, it decreased to 6,715.1 per 100,000 population by 2016. This significant reduction indicates an improvement in premature mortality rates among Whites. The temporal trend in YPLL among the Black population showed variations, with periods of decline and fluctuations. In 2000, the YPLL rate was 12,897.1 per 100,000 population, decreasing to 10,030.4 per 100,000 population by 2016. However, there were years when the YPLL rate experienced slight increases before declining again, with a rate of 9,702.3 per 100,000 population in 2015.

YPLL rates among the Asian population exhibited a consistent decline from 2000 to 2016, except for slight increases in 2014 and 2015. Starting at 3,811.1 per 100,000 population in 2000, it decreased to 3,176.8 per 100,000 population by 2016, signifying improved premature mortality rates in this group. The YPLL rates among the American Indian/Alaska Native population showed fluctuations during the study period. In 2000, the YPLL rate was 7,758.2 per 100,000 population, and it varied over the years, with no consistent declining trend observed.

**Figure 2:**
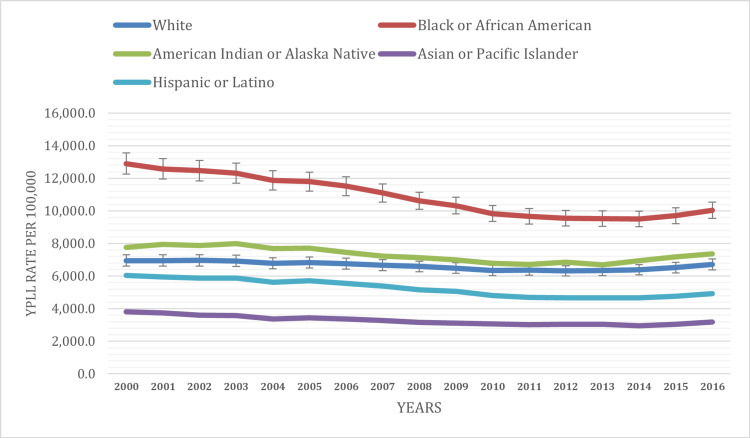
YPLL age-adjusted rate based on race

The Hispanic population experienced a notable reduction in YPLL rates over the study period. Starting at 6,037.6 per 100,000 population in 2000, it decreased to 4,926.0 per 100,000 population by 2016, indicating a significant improvement in premature mortality rates among Hispanics (Figure 2). These temporal trends underscore the disparities in premature mortality among different racial groups in the United States. While some groups experienced consistent improvements in YPLL rates, others faced fluctuations and disparities that warrant further investigation and targeted interventions.

Based on the leading cause of death

Based on the leading causes of death, the top three leading causes are malignant neoplasms (1,451.6 per 100,000 population), followed by diseases of the heart (1,055.4 per 100,000 population), and cerebrovascular diseases (182.3 per 100,000 population). Within the category of heart disease, ischemic heart disease was the most commonly reported cause of death (646.5 per 100,000 population), while the trachea, bronchus, and lung subtype of neoplasm reported a rate of 353.2 per 100,000 population (Table 2).

**Table 2:** Age-adjusted rate per 100,000 population under age 75 by leading causes of death

Age-adjusted rate per 100,000 population under age 75 for cause of death	2000	2001	2002	2003	2004	2005	2006	2007	2008	2009	2010	2011	2012	2013	2014	2015	2016
All causes	7,578.1	7,531.9	7,521.7	7,473.1	7,280.8	7,315.7	7,228.7	7,087.0	6,957.7	6,833.1	6,642.9	6,635.2	6,588.0	6,593.1	6,622.1	6,757.7	6,968.6
Cerebrovascular diseases	223.3	211.8	208.1	203.4	197.7	192.9	189.7	183.7	177.9	172.8	169.3	164.0	161.6	158.1	160.1	161.0	164.3
Diseases of heart																	
Diseases of heart	1,253.0	1,221.3	1,212.8	1,186.2	1,126.5	1,107.5	1,074.8	1,037.8	1,022.9	992.6	972.4	962.4	951.9	952.3	952.0	956.6	958.9
Ischemic heart disease	841.8	809.9	791.4	763.4	718.1	698.9	672.5	638.2	624.4	591.5	577.3	567.0	558.4	546.1	537.1	530.3	523.8
Malignant neoplasms																	
Malignant neoplasms	1,674.1	1,652.2	1,622.0	1,584.1	1,538.9	1,519.8	1,484.6	1,452.7	1,427.8	1,413.9	1,395.8	1,370.9	1,356.2	1,328.6	1,310.4	1,283.3	1,262.4
Trachea, bronchus, and lung	443.1	431.6	422.6	410.8	400.8	390.5	376.1	363.5	350.5	341.7	331.3	318.7	309.9	298.2	287.7	273.4	254.1
Colorectal	141.9	142.5	140.9	133.6	127.0	124.3	125.6	126.0	126.8	124.3	125.0	124.1	123.0	123.5	122.2	123.3	123.7
Prostate	63.6	61.8	59.8	58.2	55.3	54.4	54.1	52.7	52.2	50.1	52.2	48.5	48.1	47.5	47.6	46.7	48.3
Breast	332.6	328.1	317.2	313.4	301.6	295.4	285.9	274.0	269.2	269.6	262.4	259.4	256.0	250.0	245.9	241.9	241.0

The YPLL rates attributed to malignant neoplasms, or cancer, exhibited a declining trend during the study period. In 2000, the YPLL rate stood at 1,674.1 per 100,000 population, and it decreased to 1,262.4 per 100,000 population by 2016. This decline signifies progress in reducing premature mortality related to cancer.

**Figure 3:**
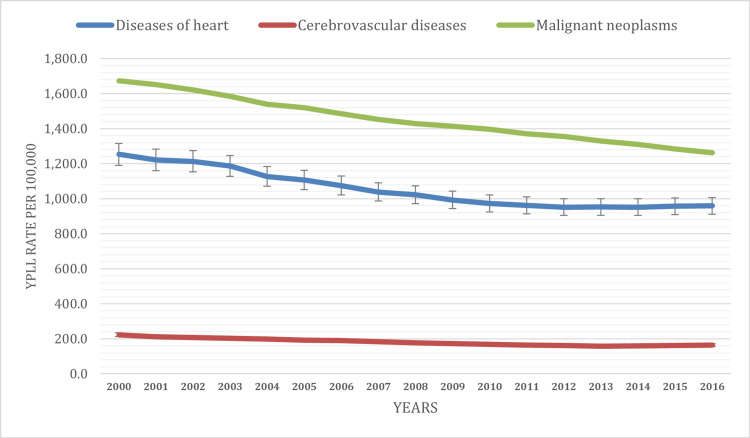
YPLL age-adjusted rate based on leading cause of death YPLL: Years of potential life lost

YPLL due to heart disease showed a substantial decline during the study period. In 2000, the YPLL rate attributed to heart disease was 1,253.0 per 100,000 population, steadily decreasing to 958.9 per 100,000 population by 2016. This consistent downward trend signifies notable progress in reducing premature mortality associated with heart disease. The temporal trend in YPLL rates due to cerebrovascular diseases demonstrated a significant decrease. In 2000, the YPLL rate for cerebrovascular diseases was 223.3 per 100,000 population, and it declined to 164.3 per 100,000 population by 2016. This decline indicates substantial improvements in preventing premature mortality associated with cerebrovascular diseases (Figure 3).

## Discussion

The findings of this comprehensive 16-year analysis of NCHS data on YPLL due to leading causes of death in the United States align with and expand upon previous research, providing valuable insights into premature mortality and health disparities. This discussion section contextualizes our findings within the existing literature, emphasizing the importance of addressing YPLL as a critical public health concern.

The sex disparities in YPLL rates align with earlier studies highlighting that males generally experience higher premature mortality rates than females. This phenomenon is attributed to differences in health behaviors, healthcare utilization, and the prevalence of specific diseases [5,10-11]. Our study reinforces the need for sex-specific health promotion strategies and interventions tailored to address the unique health challenges faced by men. The significant disparities in YPLL rates among racial and ethnic groups mirror well-documented health inequities in the United States. Our findings corroborate previous research that has identified African Americans as having the highest premature mortality rates, followed by American Indian/Alaska Native, Hispanic/Latino, and Asian/Pacific Islander populations [8,12-14]. These disparities underscore the importance of addressing the social determinants of health, healthcare access, and cultural factors contributing to health inequities.

Our study's observation of declining YPLL rates due to leading causes of death, including cancer, heart disease, and cerebrovascular diseases, is consistent with previous research. These findings affirm that public health efforts, medical advancements, and preventive measures have collectively contributed to reducing premature mortality [14-16]. The consistent downward trend in YPLL rates for heart disease, in particular, reflects the success of initiatives aimed at improving heart health [17]. The findings of this study have several public health implications. First, the identification of specific causes of death with declining YPLL rates, such as heart disease and cancer, underscores the effectiveness of public health campaigns, prevention programs, and advances in medical care. These successes should inform future efforts to reduce premature mortality further. Second, addressing the disparities in YPLL rates among demographic groups requires targeted interventions. Tailored health promotion campaigns, improved healthcare access, and culturally competent care are essential components of reducing disparities in premature mortality. Efforts should focus on understanding the unique challenges faced by different populations and developing strategies to overcome them.

One of the major strengths of this study is its extensive scope, which spans 16 years of data from the NCHS. This extended timeframe enables the identification of significant temporal trends in YPLL due to leading causes of death. Furthermore, the comprehensive nature of the NCHS data source ensures the reliability and representativeness of the findings. The study's analysis also encompasses a diverse range of causes of death, including malignant neoplasms, heart disease, and cerebrovascular diseases, providing a holistic understanding of premature mortality in the United States. Additionally, the stratification of the analysis by sex and race/ethnicity enhances the depth and applicability of the results, shedding light on disparities in YPLL across different demographic groups. This study was also associated with several limitations. First, the retrospective nature of the analysis relies on data collected for administrative purposes, which may be subject to inaccuracies or underreporting. Additionally, the study's focus on specific leading causes of death might omit other important contributors to premature mortality. Furthermore, the analysis, while stratified by sex and race/ethnicity, does not delve into potential socioeconomic factors that could influence YPLL disparities. Finally, although efforts have been made to account for trends in YPLL, causal relationships between interventions or policies and changes in YPLL are not within the scope of this analysis. These limitations highlight the need for complementary research to provide a more comprehensive understanding of premature mortality and its determinants.

## Conclusions

The declining trends in YPLL for heart disease, cancer, and cerebrovascular diseases signal progress in improving the overall health and longevity of the United States population. However, this analysis also reveals variations and fluctuations in YPLL rates, emphasizing the complex nature of health disparities. This analysis spanning 16 years highlights notable disparities in YPLL rates among different demographic groups. These differences are evident in the YPLL rates for males, which are higher than those for females. The YPLL rate is most pronounced among Black/African Americans, followed by American Indian/Alaska Natives, Hispanics/Latinos, and Asians/Pacific Islanders. The primary contributors to YPLL are malignant neoplasms, heart diseases, and cerebrovascular diseases. These findings emphasize the importance of addressing these disparities to enhance public health outcomes and mitigate the premature loss of life. Future research should delve deeper into the underlying factors contributing to these disparities, including socioeconomic determinants, access to healthcare, and lifestyle behaviors.
